# Factors Associated With Suicidal Ideation Among Persons With Disabilities in South Korea: Retrospective Observational Study

**DOI:** 10.2196/80261

**Published:** 2025-12-12

**Authors:** In-Hwan Oh, Sooyeon Jo, Joon Lee

**Affiliations:** 1 Department of Preventive Medicine, Asan Medical Center, University of Ulsan College of Medicine, Seoul, Republic of Korea; 2 Department of Preventive Medicine, College of Medicine, Kyung Hee University, Seoul, Republic of Korea; 3 Data Intelligence for Health Lab, Cumming School of Medicine, University of Calgary, 3280 Hospital Dr NW, Calgary, AB, T2N 4Z6, Canada, 1 403-220-2968; 4 Department of Cardiac Sciences, Cumming School of Medicine, University of Calgary, Calgary, AB, Canada; 5 Department of Community Health Sciences, Cumming School of Medicine, University of Calgary, Calgary, AB, Canada

**Keywords:** feature selection, machine learning, persons with disabilities, suicidal ideation, suicide attempt

## Abstract

**Background:**

South Korea has the highest suicide rate among the Organisation for Economic Co-operation and Development nations, with particularly elevated figures among persons with disabilities. Research has shown a strong correlation between suicidal ideation and suicide attempts.

**Objective:**

This study aimed to investigate the factors that contribute to suicidal ideation among persons with disabilities in South Korea, utilizing machine learning methods based on national survey data.

**Methods:**

We employed data from the 2020 National Survey on Persons with Disabilities in South Korea, which included 7025 respondents. The primary variable of interest was the answer to the question, “have you thought about taking your own life in the past year?” The dataset was divided into training (80%) and test (20%) subsets. Because the survey contained too many questions (n=1394), feature selection was conducted using random forest variable importance to identify the top 100 features. Subsequently, 5 machine learning models were trained to predict suicidal ideation based on the selected features: logistic regression, support vector machine, random forest, Extreme Gradient Boosting (XGBoost), and feed-forward neural network.

**Results:**

A total of 6832 persons with disabilities responded to the suicidal ideation question and were included in the study. The most common types of primary disability were physical disability (n=1773, 26.0%) and hearing disability (n=979, 14.3%). Of the 6832 persons with disabilities, 12.1% (n=829) indicated they had had suicidal thoughts in the past year. Significant factors that impacted suicidal ideation included intense feelings of sadness, difficulties associated with their disabilities, and overall health satisfaction. Among the models tested, the random forest model exhibited the best predictive performance with a median area under the receiver operating characteristic curve of 0.905 (IQR 0.895-0.913), a median precision of 0.592 (IQR 0.561-0.616), and a median recall of 0.588 (IQR 0.564-0.620).

**Conclusions:**

This study highlights critical predictors of suicidal ideation in persons with disabilities in South Korea, underscoring the necessity for focused mental health interventions. The results demonstrate the potential of machine learning to identify these factors, which can aid in the development of future suicide prevention strategies. Future work is warranted to investigate if the factors identified in this study are causal.

## Introduction

The suicide rate among South Koreans is so high that it ranks first in the Organisation for Economic Co-operation and Development as of 2020 [1]. It is also well known that the suicide rate among older adults is high [2], the suicide rate of persons with disabilities is higher than that of persons without disabilities, and the suicide mortality rate of persons with disabilities is more than twice that of the total population [3]. Furthermore, suicide mortality risk has been found to be pronounced in individuals with a mental disorder, visual impairment, or brain damage, particularly in those with severe forms of these disabilities [4].

Several studies have linked suicidal ideation to suicide [5]. In a study of patients receiving mental health treatment, it was reported that people with suicidal ideation were 6 times more likely to attempt suicide than those who did not, and 5 times more likely to die of suicide [6]. This is the same in South Korea, and the studies of adolescents have reported that suicidal ideation increases the risk of suicide planning by 50 times and suicide attempt by 23 times [7]. The suicide risk of people who attempted suicide was more than 20 times higher than that of the general population [8]. It has been established that suicidal ideation affects and is directly related to suicide attempts [9-11]. For example, a low-income level has been pointed out as a factor affecting suicidal ideation [12], which is also the case in South Korea [13]. Similarly, gender and age also have an effect, and it has been shown that women and older adults have higher rates of suicidal ideation [14,15].

In the case of persons with disabilities, the studies on factors affecting suicidal ideation are relatively rare, with 1 study reporting that physical disability is associated with an increased risk of lifelong suicidal ideation [16]. It is even more difficult to find a study on suicidal ideation for persons with disabilities in South Korea. Considering the high suicide rate of persons with disabilities, it is necessary to analyze the factors that affect the suicidal ideation of persons with disabilities as a starting point.

The National Survey on Persons with Disabilities (NSPD) is a survey conducted since 1980 under the Welfare of Persons with Disabilities Act to identify the proportion of the population with disabilities and their living conditions and welfare needs, and is used for policy establishment [17]. Since the NSPD collects information on the overall lives of persons with disabilities, including living conditions and welfare needs, it consists of a very large number of questions. Individual questions for each type of disability stipulated in South Korea are presented, and the common questions for persons with disabilities alone consist of more than 1000 questions. Therefore, the vastness of these data makes it difficult to identify related factors through existing statistical methods. As a way to overcome this problem, machine learning offers effective computational methods to identify important features and model the complex, nonlinear relationships between potential factors and suicidal ideation.

Therefore, this study aimed to analyze the factors affecting the suicidal ideation of persons with disabilities via the application of machine learning to the 2020 NSPD data. Supervised machine learning methods were leveraged to identify survey responses that were most strongly associated with suicidal ideation.

## Methods

### Data Source

In South Korea, disability is registered in the South Korean disability registration system, and welfare benefits are provided based on this system [17]. The 2020 NSPD data were collected from persons with disabilities registered with the Ministry of Health and Welfare as of May 2020. The NSPD dataset was constructed by taking into account gender, region, type of disability, and age, ensuring that it accurately reflects the overall characteristics of persons with disabilities in South Korea. The survey contained 1394 questions, and a total of 7025 persons with disabilities completed the survey. The response to the question “have you thought about taking your own life in the past year?” was the target variable for the machine learning models. A currency exchange rate of 1180.01 Korean Won per US dollar from 2020 was used for the household incomes reported in the NSPD [18].

### Data Partitioning

A random 20% of the dataset was held out as test data. The remaining 80% were subject to 10-fold cross-validation for training and hyperparameter tuning. All partitioning was done in a stratified manner to preserve the target variable prevalence across different datasets.

### Data Preprocessing

Of the 7025 persons with disabilities in the dataset, 193 individuals did not respond to the target variable question and were excluded. This resulted in a sample size of 6832 individuals for the subsequent steps.

Features (ie, predictor variables) with data missing rates >20% were excluded. This step excluded all 149 free-text features in the dataset and 501 continuous or categorical features. Also, the response to the question “have you attempted to take your own life in the past year?” was dropped as a feature due to its high correlation with the target variable question. In the end, a total of 742 features were included in this study.

The missing data in the included features were imputed with the median and mode for continuous and categorical features, respectively. All continuous features were scaled to be 0 mean and unit variance, whereas all categorical features were one-hot encoded. Class imbalance was mitigated by using the Synthetic Minority Oversampling Technique (SMOTE) [19] from the *imbalanced-learn* Python package with 10 nearest neighbors to equalize the sizes of the majority and minority classes. The imputation, scaling, one-hot encoding, and SMOTE were learned or executed only within the training data and subsequently applied to the validation and test data to avoid data leakage.

### Feature Selection

Since there were too many features relative to the sample size and the prevalence of the target variable, we selected the top 100 features using the *SelectFromModel* function in the scikit-learn Python package. For this feature selection, we used the feature importance measures from a random forest model with 100 trees.

### Machine Learning Modeling and Performance Evaluation

We trained and evaluated the following 5 machine learning models: logistic regression, support vector machine, random forest, Extreme Gradient Boosting (XGBoost), and feed-forward neural network. Hyperparameters were tuned via a grid search and 10-fold cross-validation. The final models were trained using the entire 80% training or validation data with the best hyperparameter settings.

Predictive performance was evaluated using the following metrics: accuracy, balanced accuracy, average precision (the area under the precision-recall curve), Brier score, *F*_1_-score, precision (positive predictive value), recall (sensitivity), and the area under the receiver operating characteristic curve (AUROC). Hyperparameter tuning optimized the mean *F*_1_-score from the 10-fold cross-validation. The test performances of the final tuned models were evaluated using 100 bootstrap samples of the test data and the metrics listed above.

All machine learning modeling was conducted using the *scikit-learn* and *xgboost* Python packages. This study followed the STROBE (Strengthening the Reporting of Observational Studies in Epidemiology) checklist for cross-sectional studies (Checklist 1).

### Ethical Considerations

This study was conducted with the approval of the Kyung Hee University Institutional Review Board (Institutional Review Board No. 21‐397 [EA]). The NSPD data are managed by the South Korean Ministry of Health and Welfare and are available to researchers for whom the need for informed consent is waived. The NSPD dataset used in this study was deidentified. Since this study was a secondary data analysis, no compensation was provided to individuals who contributed data to the NSPD. Since there are no images in this study, including in the multimedia appendices, the identification of individuals in images is not a concern.

## Results

Table 1 describes the characteristics of the 6832 individuals with disabilities included in this study. There were more male participants than female participants (n=4053, 59.3% vs n=2779, 40.7%), and the median age was 64 (IQR 52-75) years. The most common primary disability was physical disability (n=1773, 26.0%), followed by hearing disability (n=979, 14.3%), disability of brain lesion (n=805, 11.8%), and visual disability (n=786, 11.5%). Most (n=4896, 71.7%) lived in an urban area. The prevalence of having suicidal ideation in the past year was 12.1% (n=829), whereas 0.7% (n=49) of the individuals attempted suicide in the past year.

**Table 1.** Cohort characteristics of the 6832 individuals with disabilities included in this study.

Variable	Values
Sex, n (%)	
Male	4053 (59.3)
Female	2779 (40.7)
Age (y), median (IQR)	64 (52-75)
Primary disability, n (%)	
Physical disability	1773 (26.0)
Disability of brain lesion	805 (11.8)
Visual disability	786 (11.5)
Hearing disability	979 (14.3)
Speech disability	205 (3.0)
Intellectual disorder	500 (7.3)
Autistic disability	126 (1.8)
Mental disorder	374 (5.5)
Kidney dysfunction	490 (7.2)
Cardiac dysfunction	101 (1.5)
Respiratory dysfunction	144 (2.1)
Hepatic dysfunction	152 (2.2)
Facial disfigurement	75 (1.1)
Intestinal or urinary fistula	187 (2.7)
Epilepsy	135 (2.0)
Severity of disability, n (%)	
Severe	3443 (50.4)
Not severe	3389 (49.6)
Monthly household income in the past year (Won [US $]), median (IQR)	1,500,000 Won (US $1271) (800,000-2,700,000 Won [US $678-$2288])
Region, n (%)	
Large urban	3198 (46.8)
Small or medium urban	1698 (24.9)
Rural	1936 (28.3)
Overwhelming sadness or depression for 2 consecutive weeks in the past 2 years, n (%)	
Yes	1338 (19.6)
No	5494 (80.4)
Suicidal ideation in the past year, n (%)	
Yes	829 (12.1)
No	6003 (87.9)
Suicidal attempts in the past year, n (%)	
Yes	49 (0.7)
No	6783 (99.3)

Table 2 shows the predictive performances of the 5 final models on the test data, whereas the best hyperparameter settings within the search space for each model are shown in italics in Table 3. The random forest resulted in the best performance in terms of median accuracy, average precision, Brier score, precision, and AUROC. The XGBoost model resulted in the best median balanced accuracy, *F*_1_-score, and recall. Logistic regression also yielded the best median AUROC, which was tied with the random forest.

**Table 2.** Suicidal thought prediction performance on the test data.

Metric	Logistic regression, median (IQR)	Support vector machine, median (IQR)	Random forest, median (IQR)	XGBoost^a^, median (IQR)	Feed-forward neural network, median (IQR)
Accuracy	0.865 (0.861, 0.879)	0.890 (0.884-0.895)	**0.899***^**b**^* (0.895-0.904)	0.873 (0.868-0.880)	0.881 (0.875-0.888)
Balanced accuracy	0.828 (0.815, 0.839)	0.796 (0.781-0.812)	0.765 (0.753-0.781)	**0.843^b^** (0.830-0.855)	0.800 (0.784-0.809)
Average precision	0.583 (0.551-0.617)	0.609 (0.578-0.639)	**0.621^b^** (0.589-0.653)	0.599 (0.575-0.636)	0.568 (0.549-0.596)
Brier score	0.106 (0.102-0.111)	0.075 (0.071-0.079)	**0.069^b^** (0.065-0.072)	0.104 (0.100-0.108)	0.089 (0.085-0.092)
*F*_1_-score	0.587 (0.568-0.602)	0.600 (0.577-0.620)	0.590 (0.564-0.612)	**0.608^b^** (0.590-0.628)	0.586 (0.566-0.608)
Precision	0.473 (0.451-0.488)	0.537 (0.517-0.566)	**0.592^b^** (0.561-0.616)	0.491 (0.470-0.513)	0.513 (0.488-0.537)
Recall	0.781 (0.754-0.802)	0.675 (0.648-0.701)	0.588 (0.564-0.620)	**0.805^b^** (0.781-0.825)	0.693 (0.658-0.713)
AUROC^c^	**0.905^b^** (0.893-0.925)	0.897 (0.886-0.907)	**0.905^b^** (0.895-0.913)	0.896 (0.888-0.907)	0.893 (0.884-0.900)

a XGBoost: Extreme Gradient Boosting.

b The best median performance of each metric is shown in bold.

c AUROC: area under the receiver operating characteristic curve.

**Table 3.** Hyperparameter search space for each machine learning model.

Model	Hyperparameter search space
Logistic regression	penalty: l1, l2, *elasticnet^a^*, none l1_ratio: *0.5* C: 0.5, 0.6, 0.7, 0.8, 0.9, 1.0, 1.1, 1.2, *1.3*, 1.4, 1.5 class_weight: balanced, *none* Solver: *saga*
Support vector machine	kernel: linear, poly, *rbf*, sigmoid degree: 5 gamma: *scale*, auto C: *0.5*, 0.6, 0.7, 0.8, 0.9, 1.0, 1.1, 1.2, 1.3, 1.4, 1.5 class_weight: *balanced*, none
Random forest	n_estimators: 50, 100, 150, 200, *250*, 300, 350, 400, 450, 500 criterion: *gini,* entropy, log_loss specify_max_depth: *true* max_depth: *10*, 20, 30, 40, 50, 60, 70, 80, 90, 100 class_weight: balanced, *balanced_subsample*, none
XGBoost	n_estimators: 50, 100, 150, 200, 250, 300, 350, 400, 450, *500* grow_policy: 0, *1* max_depth: 10, 20, 30, 40, 50, 60, 70, 80, *90*, 100 learning_rate: 0.0001, 0.001, *0.01*, 0.1 booster: gbtree, *gblinear*, dart
Feed-forward neural network	hidden layer_sizes: (25,), (50,), (*100,*), (200,), (25, 25), (50, 50), (100, 100), (200, 200), (100, 50, 25), (200, 50, 200), (200, 100, 50, 25) activation: logistic, tanh, *relu* alpha: *0.0001*, 0.001, 0.01, 0.1 learning_rate_init: *0.0001*, 0.001, 0.01, 0.1 learning_rate: constant, *invscaling*, adaptive

a The best settings based on a grid search are shown in italics.

Table 4 shows the top 10 features used by the best model (ie, the random forest). The survey questions that the top 10 features are based on are related to the levels of depression or stress experienced, satisfaction with health status or life in general, and ability to access health care. All of the 100 selected features used by the random forest are shown in Multimedia Appendix 1.

**Table 4.** Top 10 features of the best model (random forest in Table 2) in descending order of random forest variable importance.

Survey question	Response	Random forest variable importance
In the past 2 years, have you felt overwhelmingly sad or depressed for 2 weeks in a row, to the extent that it adversely impacted your everyday life?	Yes	0.0952
In the past year, have you felt overwhelmingly sad or depressed for 2 weeks in a row, to the extent that it adversely impacted your everyday life?	No	0.0522
Do you frequently experience issues due to your disabilities?	No	0.0140
Do you frequently experience issues due to your disabilities?	Yes	0.0138
Are you currently satisfied with your life?	Slightly satisfied	0.0120
What is your assessment of your current health?	Average	0.0118
How much stress do you feel daily?	Feel a little bit of stress	0.0116
Are you satisfied with the number of friends you have?	Slightly satisfied	0.0114
In the past year, have you ever been unable to visit to a health care facility when you wanted to?	Yes	0.00869
Are you currently satisfied with your health?	Very unsatisfied	0.00815

## Discussion

### Principal Findings

In this study, we investigated the factors affecting the suicide intention of persons with disabilities by analyzing the 2020 NSPD survey data using machine learning and identifying the 100 most important factors related to suicide ideation. Whether or not the persons with disabilities had experienced depression over the past 2 years was the most important factor, followed by the question of whether they had experienced frequent problems with disability, and whether they were satisfied with their current health level, stress, the number of friends, and access to medical institutions. With respect to the prediction of suicide intention, the random forest model was found to have superiority overall, with XGBoost performing best in terms of balanced accuracy, *F*_1_-score, and recall.

Depression has been known to have a great influence on suicidal ideation [20,21]. For example, a study of Canadians aged 15‐24 years showed that depression and suicidal ideation were correlated (*r*=0.34) [22]. In South Korea, it has also been shown that depression is an important cause of increased suicidal ideation [23]. Another adolescent-focused study developed and externally validated machine learning models using survey data from South Korea, the United States, and Norway to predict suicidal thinking [24]. This study also found that feelings of sadness and despair were the most influential factor. While previous studies usually focused on the associations between depression and suicidal ideation in general populations, this study showed that this relationship is similar in persons with disabilities. In both the general and persons with disabilities populations, the relationship between depression and suicidal ideation appears to be similarly influenced by stress, satisfaction with current life, and friendship.

Whether one has experienced problems due to disability or limited access to medical institutions can be said to be relatively unique to persons with disabilities. This means that there are distinct parts of suicide ideation for persons with disabilities and that there are additional points to consider for preventing suicide. Further research is warranted to study whether improving convenience in daily life can potentially reduce the likelihood of suicidal ideation and intentions in persons with disabilities.

Conversely, what is unique in this study is that age and income level did not make the 10 most important factors. Also, gender was only ranked 64th and 72nd among the top 100 factors. This study showed that the characteristics of disabilities have an influence beyond gender, income level, or age on suicidal ideation. In other words, the characteristics of disabilities appear to be more important than other factors except for depression, and these characteristics have been identified as important factors in previous studies that analyzed suicide in persons with disabilities. For example, while both income and disability are associated with suicide, the suicide rate itself can be higher for persons with disabilities with high income levels than for people without disabilities with low-income levels [25]. This finding suggests that reducing difficulties in persons with disabilities could be beneficial in lowering the suicide rate and reducing the gap between persons with disabilities and people without disabilities, although it is difficult to draw a definitive conclusion based on the results of this observational study.

In South Korea, individuals with a disability are required to be registered to access welfare benefits, and registration is divided into 15 main types of disability. Disabilities are primarily classified as physical and mental disorders and include 6 external physical dysfunctions (physical, brain lesion, visual, hearing, speech, and sleep disorders) and 6 internal organ disorders (renal, heart, liver, respiratory, ostomy, and epilepsy). Mental disorders consist of 2 developmental disorders (intellectual and autistic disorders) and psychiatric impairments. In this study, the type itself was found to be less important with respect to suicidal ideation. Previous research has shown that individuals with mental disorders have a higher suicide rate. However, for persons with disabilities as a whole, the discomfort or barriers they face appear to be an even more significant risk factor [25]. Therefore, reducing environmental barriers for persons with disabilities may be a top priority, not only for those with mental disorders but for all individuals with disabilities.

For example, in South Korea, a primary care physician program for persons with disabilities has recently been implemented [26]. This system takes a comprehensive approach to address the barriers to accessing medical care experienced by individuals with a disability. Although it is still struggling with low participation rates, this approach can help ease the difficulty of accessing medical institutions due to disability.

Machine learning proved to be effective in this study in identifying the most important features associated with suicidal ideation in a complex, multidimensional dataset where a total of 742 features from 6832 individuals were investigated. Extensive hyperparameter tuning on 5 different machine learning models representing a range of model complexity and the use of a class imbalance mitigation technique (ie, SMOTE) led to robust results. The ability of machine learning to learn nonlinear multivariable relationships in an empirical way without the need to make assumptions about underlying data distributions was well suited to the dataset used in this study.

### Limitations

The limitations of the study can be pointed out as follows. First, this study is a cross-sectional observational study, and causation cannot be established. Although the results of this study are largely supported by the literature, further research is needed to investigate causal relationships. Second, this study included only registered individuals with a disability. There are unregistered persons with disabilities who were excluded. Finally, while we investigated reasonably diverse machine learning methods in this study, there are other feature selection and class imbalance mitigation methods, as well as other machine learning models, that may have led to better prediction performance.

### Conclusions

This study makes novel contributions by identifying factors associated with suicidal ideation using a large survey dataset collected from persons with disabilities in South Korea. The use of machine learning was another novel aspect of this study. Our results showed that both disability characteristics such as general discomfort caused by the disability and nondisability characteristics such as depression are important factors. Future work can focus on studying whether the associations between these factors and suicidal ideation are causal.

## Supplementary material

10.2196/80261 Multimedia Appendix 1 List of 100 selected features.

10.2196/80261 Checklist 1 STROBE checklist.

## Abbreviations

AUROC: area under the receiver operating characteristic curve
NSPD: National Survey on Persons with Disabilities
SMOTE: Synthetic Minority Oversampling Technique
STROBE: Strengthening the Reporting of Observational Studies in Epidemiology
XGBoost: Extreme Gradient Boosting
